# Probabilistic ranking of plant cultivars: stability explains differences from mean rank

**DOI:** 10.3389/fpls.2025.1553079

**Published:** 2025-03-20

**Authors:** Shayan Tohidi, Sigurdur Olafsson

**Affiliations:** Department of Industrial and Manufacturing Systems Engineering, Iowa State University, Ames, IA, United States

**Keywords:** cultivar selection, environmental uncertainty, G×E effects, probabilistic comparison, stability

## Abstract

An alternative to ranking cultivars based on mean and stability of phenotype is evaluating pairs of cultivars and for each pair estimating which cultivar is more likely to perform better across a random subset of target environments. Such pairwise probabilistic order can then be translated into probabilistic ranking of all cultivars that accounts for both mean and stability in a single metric. Mean and probabilistic order will be the same for most cultivar pairs; but the pairs that differ reflect differences in stability and should thus be at least partially explained by existing stability measures. We formulate a classification problem to predict differences between mean and probabilistic order for a pair of cultivars with the predictor variables defined as differences in stability. We then apply a feature selection method to identify the best predictors, that is, the stability measures that are most predictive of the differences in the two orders. The results from applying this method to data observed from multiple crops, namely, rapeseed, sorghum and maize, show that a) existing stability measures explain most of the differences, b) no stability measure explains all differences for all data, and c) stability measures that combine mean with specific type of stability perform the most like probabilistic order. These results support the premise that probabilistic ranking combines mean and stability; but no existing stability measure can completely replace estimating the relevant probabilities to identify the cultivars that are more likely to perform better across their target environments.

## 1 Introduction

This paper addresses how probabilistic ranking of cultivars can account for both mean and stability of phenotypic performance of the crops. Similar ideas have been proposed before but the literature is sparse. Westcott (1986) is the earliest survey to observe that applying probabilistic ideas to analyze genotype-byenvironment (G×E) interactions is potentially useful for plant breeding. But despite substantial very early work (Anderson, 1974; Barah et al., 1981; Byth et al., 1976; Menz, 1980), relatively little has still been done to bring probabilistic ideas into plant breeding practice. A more common practice for plant breeders is to consider mean performance, e.g., select the cultivar with the highest yield as determined by some statistical model, and enhance this with G×E analysis based on the statistical model or other measures of stability or adaptability (Gauch, 2006; Gauch et al., 2008; Piepho et al., 2008). Combining mean and some measure of variability in a single measure has also been proposed in different ways by numerous researchers (Piepho, 1998).

The environmental effect (E) and the G×E interaction effects are both major contributors to the phenotype observed for most food crops. Thus, when comparing different cultivars of those crops with the goal of differentiating the genetic effect (G) of cultivars, the uncertainty due to the set of environments observed is substantial. A *probabilistic alternative* for plant breeders faced with selecting one cultivar over another is to view the environments as random and select cultivars that are *most likely* to perform best across a set of target environments rather than select the cultivar with the highest mean. An approach that accounts for the underlying probability distribution of the environments combines the mean performance with the risk due to planting environments and their interaction with the cultivars being tested. From a utility theory perspective, this may be considered maximizing the utility for a risk-averse decision-maker, which is similar to what has been done in other agricultural applications (DeVuyst and Halvorson, 2004; Liu et al., 2017; Stanger et al., 2008).

While stochastic dominance has received substantial attention in agricultural economics (Levy, 1992), such probabilistic approaches have received much less attention in plant breeding. Some notable exceptions include work that uses a probabilistic approach to select low-risk cultivars and compare experimental cultivars against a check (Eskridge, 1990; Eskridge and Mumm, 1992) and recent work that suggests a more comprehensive Bayesian approach to accounting for G×E interactions (Dias et al., 2022) with similar goals. Another related approach is considering weight for yield and stability measures to find the genotypes with highest yield and lowest risk (Eskridge et al., 1991). We will draw directly on similar work that studies probabilistic orders, where the underlying probabilities are estimated via bootstrap sampling of planting environments (Bijari, 2022). In this work the uncertainty that is accounted for is the uncertainty due to the subset of environments observed, which is the approach we adopt.

The intuitive reason for why mean and probabilistic order might differ is that the distribution of phenotype difference may be skewed when the underlying uncertainty is due to planting environment (as opposed to the uncertainty within each environment). For example, a cultivar that does extremely well in very favorable environments might have a better mean than a cultivar that performs more evenly across all environments because a few extreme phenotype values have a large effect on the mean. But the cultivar with the worse mean may be more likely to perform better in a random environment, or a subset of random environments, because the very favorable environments occur with a low probability. This paper aims to explore and explain those differences in more depth using a comprehensive set of known stability measures, thus providing a case for why plant breeders may want to consider a probabilistic approach when ordering and ranking cultivars based on phenotypic response.

To evaluate the premise that selecting the cultivar that is more likely to perform better in a set of target environments accounts for both the stability across environments and mean performance, we address whether the differences between mean and such probabilistic order for a pair of cultivars can be explained by one or more of the many stability measures proposed in the literature. We hypothesize that such measures partially explain these differences but that no existing stability measure explains all the differences in rank. We further hypothesize that some stability measures explain more of those differences than others. The paper thus addresses the following questions: Are the differences in mean versus probabilistic pairwise order completely explained by existing stability measures? And are there specific types of stability measures that are the best predictors of those differences?

Using an analysis of plant breeding data of three different crops (rapeseed, sorghum and maize), the study show that the mean and probabilistic rank is highly correlated, suggesting that the pairwise orders are usually the same. Some stability measures are also highly correlated with probabilistic rank. This is especially true for measures that directly combine both mean performance and some type of stability, which supports the assertion that the probabilistic ranking combines mean performance and stability into a single metric. When mean and probabilistic order is different, the results show that those differences are explained by some stability measure, but depending on the data they are best explained by different stability measures, and no measure can simply replace probabilistic comparison and account for all the differences.

## 2 Materials and methods

We first set up a classification problem where there is a binary dependent variable to be predicted based on multiple independent variables. The dependent variable here is an indicator function that shows if a pairwise order of two cultivars is the same or not for mean versus probabilistic order; that is, for that pair, is the cultivar with the higher mean also the cultivar that is more likely to perform better? We take the independent variables as a similar pairwise order using a comprehensive set of known stability measures. If stability measures explain the differences between mean and probabilistic order, the dependent variable should be predictable using the independent variables. We then propose a feature selection method to identify the best predictors, that is, a method to determine which stability measures best explain the differences in order. Finally, we apply this classification formulation and feature selection method to plant breeding data involving rapeseed, sorghum and maize.

### 2.1 Notation and probabilistic comparison

We assume that there are *n* cultivars that have been planted in the same set *E* of environments. To rank the cultivars we need to be able to order any pair and then apply arbitrary ranking algorithm to find a complete rank. We thus focus on a pair (*i, j*) of cultivars where *i, j* ∈ {1, 2*,…, n*} and *i < j*. For each of these cultivars we have a set of phenotype observations *y_i_* (*e*) and *y_j_* (*e*), ∀*e* ∈ *E*. Based on the observations we define two indicator functions:


$$I^{\left(P\right)}\left(i,j\right)=\{\begin{array}{ll} 1, & \text{if cultivar }i\text{ is more likely to outperform }j\text{ in a random subset of }E,\\ 0, & \text{otherwise}; \end{array}$$

$$I^{\left(M\right)}\left(i,j\right)=\{\begin{array}{ll} 1, & \text{if cultivar }i\text{ has better mean performance than }j\text{ in }E,\\ 0, & \text{otherwise}. \end{array}$$

The second indicator $I^{\left(M\right)}\left(i,j\right)$ can be easily estimated from the observed data as the simple mean difference across all the environments:


$${\hat{I}}^{\left(M\right)}\left(i,j\right)=\{\begin{array}{ll} 1, & \text{if }\frac{1}{\left|E\right|}\sum_{e\in E}y_{i}\left(e\right)-y_{j}\left(e\right)>0,\\ 0, & \text{otherwise}. \end{array}$$

Obtaining an estimate of $I^{\left(P\right)}\left(i,j\right)$ requires more work. One approach that has been proposed is to use bootstrap resampling of environments to estimate the underlying probabilities, that is, $P(Y_{i}>Y_{j})$ , where *Y_i_* is a random variable describing the phenotype of cultivar *i* in a random subset of environments (Bijari, 2022). The bootstrap estimate $\hat{P}(Y_{i}>Y_{j})$ can then be used to estimate the indicator accordingly:


$${\hat{I}}^{\left(P\right)}\left(i,j\right)=\{\begin{array}{ll} 1, & \text{if }\hat{P}(Y_{i}>Y_{j})>\frac{1}{2},\\ 0, & \text{otherwise}. \end{array}$$

In this paper we simply use this approach, that is, repeatedly sample environments with replacement to obtain a bootstrap estimate $\hat{P}(Y_{i}>Y_{j})$ of the needed probabilities and then use that to estimate the indicator as shown above. Bijari (2022) showed that while ${\hat{I}}^{\left(P\right)}\left(i,j\right)=\;{\hat{I}}^{\left(M\right)}\left(i,j\right)$ for most cultivar pairs, they differ in important cases. Specifically, if the difference in the main effect is small, the G×E structure of the two cultivars differ, and the magnitude of the G×E effects is large, which makes it more likely that ${\hat{I}}^{\left(P\right)}\left(i,j\right)\neq \;{\hat{I}}^{\left(M\right)}\left(i,j\right)$ . Thus, it is plausible that at least some of the differences may be explained by traditional stability measures.

### 2.2 Stability measures

As predicting the mean and then either complementing or combining the mean rank with one or more measures of stability is perhaps a more intuitive, and certainly more familiar, alternative to a probabilistic rank, it is of interest to understand if known stability measures provide sufficient information to supplement mean order; or in other words, if such measures explain the differences between mean and probabilistic order. To that end, we will consider a selection of 39 stability measures proposed in the literature. All the measures are applied using the *metan* R package (Olivoto and Lúcio, 2020), which implements a comprehensive set of stability measures. Note that these measures include both measures of what has been termed static and dynamic stability, as well as those that have elements of both. Specifically, we consider the following metrics: variance (*Var*), coefficient of variation (*CV*), adjusted coefficient of variation (*ACV*) (Döring and Reckling, 2018), power law residuals (*POLAR*) (Döring et al., 2015), Shukla’s variance (*Shukla*) (Shukla, 1972; Kang and Pham, 1991), Annicchiarrico’s confidence index (*Wi_g_*, *Wi_f_*, *Wi_u_*) (Annicchiarico, 1992), Wricke’s ecovalence (*Ecoval*) (Wricke, 1965), deviations (*S_ij_*) and R-squared (*R_2_*) from the joint-regression analysis (Eberhart and Russell, 1966), AMMI based stability parameter (*ASTAB*) (Rao and Prabhakaran, 2005), AMMI stability index (*ASI*) (Jambhulkar et al., 2017), AMMI stability value (*ASV*) (Purchase et al., 2000), sum across environments of absolute values of GEI modeled by AMMI (*AVAMGE*) (Zali et al., 2012), Annicchiarico’s D parameter values (*Da*) (Annicchiarico, 1997), Zhang’s D parameter (*Dz*) (Zhang et al., 1998), sums of the averages of the squared eigenvector values (*EV*) (Zobel, 1994), stability measure based of fitted AMMI model (*FA*) (Raju, 2002), modified AMMI stability index (*MASI*) (Ajay et al., 2018), modified AMMI stability value (*MASV*) (Ajay et al., 2019), sums of absolute value of the IPC scores (*SIPC*) (Sneller et al., 1997), absolute value of the relative contribution of IPCs to the interaction (*Za*) (Zali et al., 2012), weighted average of absolute scores for BLUP analysis (*WAASB*) (Olivoto et al., 2019; Möhring et al., 2015), harmonic mean of the genotypic value (*HMGV*), relative performance of the genotypic value (*RPGV*), and harmonic mean of the relative performance of the genotypic value (*HMRPGV*) (Smith et al., 2005; Alves et al., 2018; Azevedo Peixoto et al., 2018; Colombari Filho et al., 2013; Dias et al., 2018), superiority index (*Pi_a_*, *Pi_f_*, *Pi_u_*) (Lin and Binns, 1988), geometric adaptability index (*GAI*) (Mohammadi and Amri, 2008), Huehn’s stabilities (*S_1_*, *S_2_*, *S_3_*, *S_6_*) (Huehn, 1979), and Thennarasu’s stabilities (*N_1_*, *N_2_*, *N_3_*, *N_4_*) (Thennarasu, 1995).

### 2.3 Correlation of rank

For an overall assessment of if the rank produced by the probabilistic order is different than rank produced by either just the simple mean phenotype or the stability according to one of the 39 stability measures considered, we calculate the rank correlation of different ranks. Specifically, we use Spearman’s rank correlation and perform a statistical test to determine if this rank correlation *ρ* ^Rank^ indicates that the ranks are different. The null hypothesis is thus that the ranks do not have positive correlation, that is *H*_0_: *ρ* ^Rank^ ≤ 0 versus the alternative *H*_1_: *ρ* ^Rank^ *>* 0.

### 2.4 Predicting the difference between mean and probabilistic order

The primary goal of this work is to determine the extent to which one of the 39 existing stability measures considered explain the differences between mean and probabilistic order. That is, we want to determine if the probabilistic order could also be captured by the more familiar approach of considering mean order supplemented with one or more stability measures. Our approach to answer this question is to formulate a classification problem and use this classification formulation to evaluate the predictability of the differences.

We use pairwise orders to express the differences between probabilistic and mean order. In this method, instead of directly contrasting two ranked lists for all cultivars, each cultivar pair is compared by using their probabilistic and mean pairwise order. Thus, each pair of cultivars is labeled as having either the same order or different order using the two methods. This defines the class or dependent variable as follows:


$$y_{ij}=\{\begin{array}{ll} 0, & \text{if }{\hat{I}}^{\left(P\right)}\left(i,j\right)={\hat{I}}^{\left(M\right)}\left(i,j\right),\\ 1, & \text{otherwise}. \end{array}$$

The predictor or independent variables are given by a comparison of m = 39 stability measures:


$$△s_{ij}^{\left(k\right)}=s_{i}^{\left(k\right)}-s_{j}^{\left(k\right)}\;\text{∀}k\in \left\{1,2,\ldots ,m\right\},$$

where $s_{i}^{\left(k\right)}$ and $s_{j}^{\left(k\right)}$ are the results of calculating stability measure *k* on cultivars *i* and *j*, respectively. Each data point is now given by $\left(\Delta g_{ij},s_{ij}^{\left(1\right)},\ldots ,s_{ij}^{\left(m\right)},y_{ij}\right)$ , where *g_i_* is the mean phenotype of cultivar *i* across all the environments and △*g_ij_* = *g_i_* − *g_j_* is their difference between genotypes *i* and *j*. The number of observations in one dataset depends on the number of cultivars and is given by $\frac{n\left(n-1\right)}{2}$ , where *n* is the number of cultivars. Constructing such data points for each pair results in a dataset that can be used to predict when ${\hat{I}}^{\left(P\right)}\left(i,j\right)\neq \;{\hat{I}}^{\left(M\right)}\left(i,j\right)$ , that is, when probabilistic and mean order result in different pairwise selection.

For demonstration purposes, we illustrate two partial rows of a constructed training data for this classification problem. This is constructed using rapeseed data (Shafii and Price, 1998) that will also be used in the results section below.

**Table:** The first two partial rows of the training data according to rapeseed dataset.

(i,j)	$△g_{ij}$	$△s_{ij}^{\left(1\right)}$	$△s_{ij}^{\left(2\right)}$	…	$△s_{ij}^{\left(m\right)}$	$y_{ij}$
(Bienvenu,Bridger)	118.34	19.63	0.28	…	-0.15	1
(Bienvenu,Cascade)	165.42	10.74	0.14	…	-0.15	0
⋮	⋮	⋮	⋮		⋮	⋮

The details of this data are described below, but for the above it suffices to recognize that Bienvenu and Bridger are two of the rapeseed cultivars in this data and the phenotype is yield with units of kg/ha. The first row above shows that for Bienvenu and Bridger the difference in yield is 118.34 kg/ha, so Bienvenu is better according to mean order; whereas the label *y_ij_* = 1 implies that Bridger is more likely to perform better than Bienvenu across the set of target environments, that is, the mean and probabilistic orders differ. These and all other labels are obtained by comparing the mean difference found in the original data with the bootstrap estimates of the probabilities. Values for the independent variables are found by calculating the stability measures for each cultivar using the *metan* R package (Olivoto and Lúcio, 2020).

### 2.5 Value of each stability measure

The features or predictive variables in our classification model are the differences in the stability measures. Thus, if those stability measures explain the differences in mean versus probabilistic order (the class variable) then it should be possible to construct an accurate classification model. As predicting those differences is secondary to determining which stability measures are the best predictors, we focus on what is called feature selection, that is, to determine which stability measure differences are the best predictors of when the probabilistic order differs from a mean order.

Many standard feature selection methods have been proposed but we design a method specifically for this problem. The motivation for an application-specific method is that while evaluating the stability measures directly as predictor variables is expected to provide some insights, it is unlikely that any stability measure is highly predictive on its own because it is expected that there is a strong relationship between the differences being predicted and the mean difference. Specifically, if the mean difference in phenotype is large enough, then the mean and probabilistic order should always be the same. Thus, combining mean differences and the stability measure would be more likely to explain the differences.

**Figure 1:**
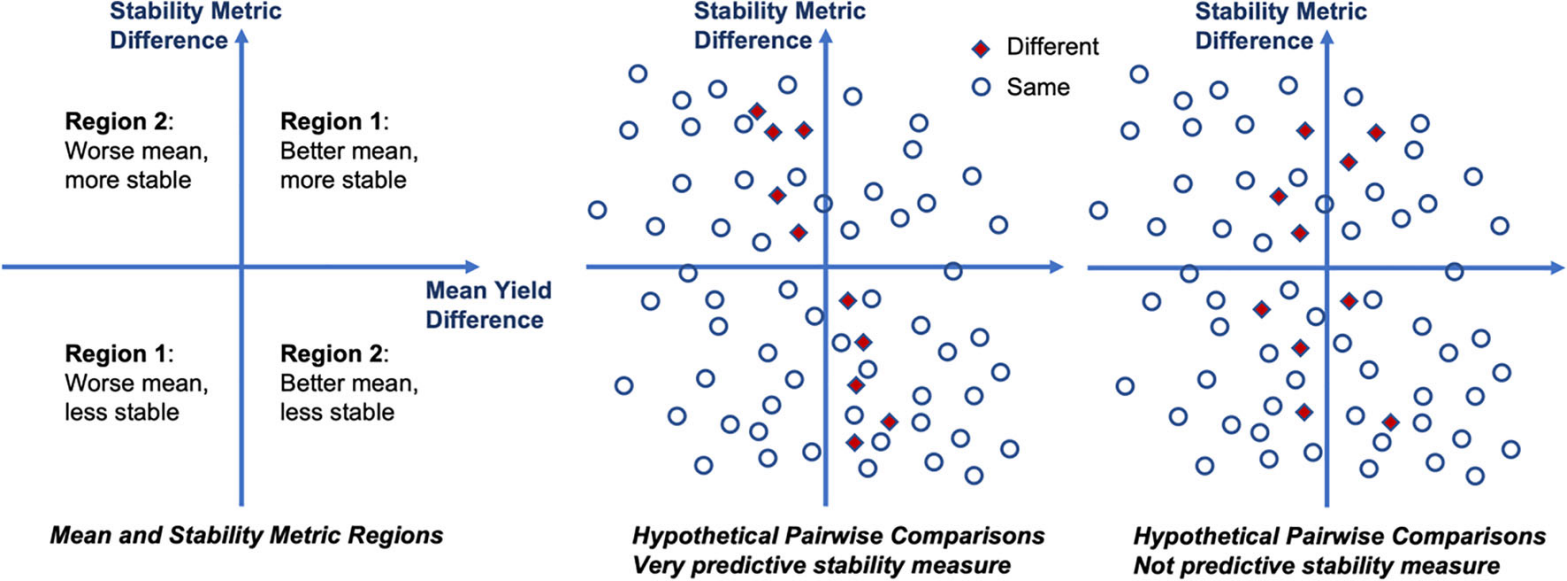
Illustration of measuring the quality of stability measures. If the stability measure difference explains the differences in order, it is either because the stability measure is better even though the mean is worse (top-left quadrant), or because less stability outweighs better mean (bottom-right quadrant). Thus, if different pairwise orders are concentrated in those two quadrants (region 2), then we find this stability measure to be predictive of the differences.

The idea is visualized in **Figure 1**. In each of the plots in this figure the *x*-axis shows the mean phenotype difference between two cultivars, and the *y*-axis shows the stability measure difference for the same pair. We define two regions based on the four quadrants in each plot by combining the diagonal quadrants into one region. The intuitive motivation for this is that if the stability metric explains all the differences, then those differences would all be in the same region. The null hypothesis is thus be that data points are placed in each region with the same relative frequency, so it can be concluded that the data points are distributed uniformly in those regions. In other words, it tests a null hypothesis that the joint distribution in the following contingency table is the product of its row and column marginals.

**Table:** The contingency table of the classification problem.

	Region 1 ( j=1 )	Region 2 ( j=2 )	Total
Class: Different ( i=1 )	$x_{11}$	$x_{12}$	$n_{1}=x_{11}+x_{12}$
Class: Same ( i=2 )	$x_{21}$	$x_{22}$	$n_{2}=x_{21}+x_{22}$
Total	$x_{11}+x_{21}$	$x_{12}+x_{22}$	$n_{1}+n_{2}$

Here *x_ij_* indicates the number of data points of class *i* falling into region *j*, *n_i_* = *x_i_*_1_ + *x_i_*_2_ shows the number of data points of class *i*, and ${\hat{p}}_{j}=\frac{x_{1j}+x_{2j}}{n_{1}+n_{2}}$ is the proportion of data points falling into region j. A test statistic can be defined by looking at the squared differences as follows:


$$\chi^{2}=\sum_{i=1}^{2}\sum_{j=1}^{2}\frac{{\left(x_{ij}-n_{i}{\hat{p}}_{j}\right)}^{2}}{n_{i}{\hat{p}}_{j}}.$$

This statistic follows a chi-squared distribution, and if the *p*-value is less than or equal to the significance level (*α* = 0.05), we reject the null hypothesis and conclude that the stability measure is statistically significant and appears to explain at least some of the differences between the mean and probabilistic order.

### 2.6 Data

#### 2.6.1 Rapeseed and sorghum data

We analyze two small datasets used for stability analysis in past work, one for rapeseed yield and one for sorghum yield, both obtained from the *Agridat* R package (Wright, 2024). Analyzing small datasets allows us to evaluate and explain each instance of different orders between the two approaches. There are important differences for both datasets due to large G×E effects; that is, the differences between the mean-based and probabilistic order are meaningful. A key difference between the two datasets is that the rapeseed data has few genotypes (6) versus environments (27), whereas the sorghum data has relatively many genotypes (18) versus environments (6). These two datasets thus provide a reasonable basis for an initial analysis.

The rapeseed data was reported by Shafii and Price (1998) and describes 6 rapeseed cultivars (Bienvenu, Bridger, Cascade, Dwarf, Glacier, and Jet) in 27 environments (14 locations and 3 years). The mean yield, mean and probabilistic ranks, and the relative yield of each cultivar in each environment are shown in **Table 1**. It is apparent from the relative performance in each environment that there are significant G×E effects. From the ranks, we note that the mean and probabilistic ranks are different for the top three cultivars.

**Table 1:** Mean yield, ranks, and relative yield in each environment for the rapeseed data.

		Genotypes					
		Bienvenu	Glacier	Bridger	Cascade	Jet	Dwarf
	Mean Yield	2487.95	2481.78	2369.61	2322.53	2278.76	2268.98
	Mean Rank	1	2	3	4	5	6
	Prob. Rank	3	1	2	4	5	6
	Unit	Kh/ha					
Yield Relative to Environmental Mean	GGA87	46.24	284.43	-32.79	108.45	-305.16	-101.16
	GGA89	158.47	412.35	-390.77	44.14	-36.85	-187.33
	ID87	230.70	99.58	-64.78	-238.17	52.58	-79.90
	ID88	1391.21	-17.54	-573.29	-260.04	-388.04	-152.29
	ID89	-45.72	163.58	-1238.68	91.13	470.30	559.40
	KS88	58.96	148.21	-28.29	-229.04	-38.04	88.21
	MS88	251.54	656.89	273.96	-605.89	-338.25	-238.24
	MT87	-513.92	176.83	-301.67	-213.67	530.58	321.83
	NC87	-120.31	108.07	-56.98	-375.87	552.51	-107.42
	NC89	122.98	139.5	359.48	-156.97	-557.41	92.43
	NY89	-205.15	162.65	-9.24	-552.63	97.96	506.42
	OR87	929.55	558.82	-1096.47	-290.55	-406.84	305.49
	OR88	722.65	-199.85	-359.29	-681.55	220.49	297.55
	SC87	38.54	256.04	243.79	-342.46	371.54	-567.5
	SC88	33.03	490.51	110.67	-120.82	-451.36	-62.04
	SC89	2.74	-127.6	1034.33	952.43	-911.74	-950.2
	TGA87	-94.41	101.04	515.5	355.64	-398.19	-479.6
	TGA88	-1.21	363.92	201.95	314.88	-382.32	-497.2
	TGA89	-309.32	-222.17	1006.61	721.06	-439.63	-756.6
	TN89	421.08	-278.93	-58.18	-537.15	309.11	144.06
	TX87	-160.33	-46.58	70.67	-263.58	409.92	-10.08
	TX88	345.34	-166.18	-21.58	201.7	-23.01	-336.3
	VA88	-313.17	-63.70	263.72	40.09	44.55	28.51
	VA89	-2.24	226.89	-32.29	-221.06	121.07	-92.37
	WA87	-41.88	323.13	186.88	47.38	-72.63	-442.88
	WA88	1682.38	-149.88	-892.88	163.38	-846.88	43.88
	WA89	-1396.25	-335.25	925.75	814.25	-1.00	-7.50

The sorghum data was reported by Omer et al. (2015). This dataset contains 432 observations of 18 cultivars in 6 environments (2 locations across 3 years). There are four replications in randomized complete block design in each environment. Like the rapeseed data, the mean yield, mean and probabilistic ranks, and the relative yield of each cultivar in each environment for the sorghum data are shown in **Table 2**. Again, we note that significant G×E effects are apparent from the relative performance. While there are many similarities between the two ranks (e.g., the bottom seven cultivars are the same for both ranks), there are large differences in rank for many cultivars.

**Table 2:** Mean yield, ranks, and relative yield in each environment for the sorghum data.

	Mean Yield	Rank		Yield Relative to Environmental Mean Yield						
		Mean	Prob	E1	E2	E3	E4		E5	E6
Unit	Kg/ha			Kg/ha						
**G13**	619.57	1	2	-47.94	333.23	153.88		115.58	149.54	36.42
**G15**	591.96	2	4	-121.98	130.67	-24.57		6.01	19.25	565.72
**G10**	591.06	3	1	142.32	-113.80	67.37		183.67	206.94	83.18
**G09**	544.85	4	5	91.24	129.42	142.01		-122.35	-76.21	128.29
**G14**	543.67	5	8	-43.66	13.64	-88.23		-74.00	12.23	465.33
**G18**	534.36	6	11	30.19	-50.05	358.98		195.42	-113.45	-191.63
**G08**	522.54	7	3	-21.63	172.27	70.23		-56.25	-60.56	54.47
**G11**	522.07	8	6	32.77	43.48	56.99		164.98	19.22	-161.70
**G17**	521.50	9	10	-124.49	-16.73	-385.95		521.04	19.22	139.23
**G02**	507.85	10	7	38.22	20.65	-155.81		164.46	-33.52	36.42
**G12**	500.95	11	9	13.02	195.67	-132.17		-179.89	-22.14	154.54
**G07**	500.37	12	14	-107.71	-121.67	265.91		48.91	-32.35	-27.56
**G05**	458.27	13	13	12.93	-205.08	-28.24		-18.40	15.94	-4.19
**G03**	428.72	14	12	15.01	-98.30	133.78		-202.89	29.95	-281.92
**G16**	403.83	15	15	43.81	-41.64	-66.49		-154.64	-69.32	-265.45
**G04**	398.81	16	18	41.27	-181.58	78.11		-81.78	-70.06	-369.75
**G01**	380.50	17	16	-13.72	-67.47	-89.29		-181.20	5.52	-347.49
**G06**	359.16	18	17	20.31	-142.71	-356.50		-328.68	-0.24	-13.89

#### 2.6.2 Maize datasets

For larger data to validate the results and the insights obtained on the rapeseed and sorghum data, we utilize datasets constructed from the Genome to Field (G2F) project (Lawrence-Dill et al., 2019). These are a rich source of observations for maize crops and include multi-year, multi-environment phenotypic evaluations. We take all the phenotypic datasets from 2014 to 2022 as the starting point and then do cleaning and pre-processing to construct complete datasets, that is, datasets where each maize hybrid is observed in all environments with more than one repetition. These results exist in 43 datasets with the number of environments between 5 and 48, the number of cultivars between 6 and 84, and the number of observations between 98 and 1770 in each dataset and 3590 total observations across 42 locations in 8 years and 738 maize hybrids. A description of the 43 datasets can be found in **Table 3**. The original datasets and more information about them are available at https://github.com/ShayanTohidi/.

**Table 3:** Description of Maize Datasets constructed from the Genome to Field Data.

Name	Hybrids	Environments	Locations	Year(s)
**Maize1**	84	6	6	2014
**Maize2**	48	6	6	2014
**Maize3**	32	7	7	2014
**Maize4**	8	12	12	2014
**Maize5**	12	8	8	2014
**Maize6**	21	27	27	2015
**Maize7**	7	7	7	2015
**Maize8**	21	16	16	2016
**Maize9**	24	13	13	2016
**Maize10**	22	14	14	2016
**Maize11**	17	17	17	2016
**Maize12**	7	15	15	2016
**Maize13**	47	10	10	2016
**Maize14**	51	8	8	2016
**Maize15**	19	28	17	2016, 2017
**Maize16**	17	29	17	2016, 2017
**Maize17**	7	33	17	2016, 2017
**Maize18**	32	21	11	2016, 2017
**Maize19**	44	18	11	2016, 2017
**Maize20**	24	23	11	2016, 2017, 2018
**Maize21**	59	15	15	2017
**Maize22**	29	16	16	2017
**Maize23**	7	27	27	2017
**Maize24**	61	12	12	2017
**Maize25**	63	11	11	2017
**Maize26**	9	10	10	2017
**Maize27**	65	10	10	2017
**Maize28**	50	17	12	2017, 2018
**Maize29**	7	16	12	2017, 2018
**Maize30**	40	13	11	2017, 2018
**Maize31**	6	12	11	2017, 2018
**Maize32**	57	6	6	2018
**Maize33**	68	5	5	2018
**Maize34**	9	23	23	2020
**Maize35**	11	7	7	2020
**Maize36**	8	48	27	2020, 2021
**Maize37**	8	8	8	2021
**Maize38**	7	7	7	2021
**Maize39**	7	7	7	2021
**Maize40**	16	25	25	2021
**Maize41**	30	14	14	2022
**Maize42**	25	8	8	2022
**Maize43**	7	26	26	2022

## 3 Results

### 3.1 Correlation of stability and probabilistic rank

**Table 4** compares probabilistic rank with both mean rank and rank according to four stability statistics (*Shukla*, *N_2_*, *GAI*, and *Pi_a_*) for the rapeseed data. **Table 5** shows the same comparison for the sorghum data. We observe that for both data the probabilistic ranks are highly correlated with mean ranks (*ρ* ^Rank^ is close to 1), while the correlation with stability metrics ranges from close to -1 to 1; and most of the time, their correlation is less than that with mean ranks. This illustrates that the mean yield plays a dominant role in probabilistic ranking.

**Table 4:** Comparison of probabilistic rank versus mean rank and ranks according to four stability measures: *Shukla*, *N_2_*, *GAI*, and *Pi_a_* for the rapeseed data.

Cultivar	Rank						
	Prob.	Mean	Stability Metrics				
			*Shukla*		*N_2_*	*GAI*	*pi_a_*
Glacier	1	2	1	6		1	2
Bridger	2	3	5	5		2	5
Bienvenu	3	1	6	4		3	1
Cascade	4	4	4	2		4	3
Jet	5	5	3	3		5	6
Dwarf	6	6	2	1		6	4
*ρ* ^Rank^		0.829	-0.086	-0.943		1	0.429
*p*-value		0.029	0.599	0.999		0.001	0.209

**Table 5:** Comparison of probabilistic rank versus mean rank and ranks according to four stability measures: *Shukla*, *N_2_*, *GAI*, and *Pi_a_* for the sorghum data.

Cultivar	Rank					
	Prob.	Mean	Stability Metrics			
			*Shukla*	*N_2_*	*GAI*	*pi_a_*
G10	1	3	6	18	1	3
G13	2	1	8	16	2	1
G08	3	7	2	11	6	6
G15	4	2	17	14	12	2
G09	5	4	7	17	4	5
G11	6	8	4	9	3	8
G02	7	10	3	7	5	7
G14	8	5	16	3	8	4
G12	9	11	11	10	7	12
G17	10	9	18	15	17	11
G18	11	6	15	13	9	10
G03	12	14	12	8	10	14
G05	13	13	1	2	11	13
G07	14	12	10	6	16	9
G16	15	15	5	1	13	15
G01	16	17	9	4	14	17
G06	17	18	14	12	18	18
G04	18	16	13	5	15	16
*ρ* ^Rank^		0.905	0.230	-0.643	0.820	0.913
*p*-value		0	0.178	0.998	0	0

We repeat the same calculation for each of the remaining 35 stability metrics and identify the stability metrics where we cannot reject the null hypothesis that rank correlation equal one at a 95% confidence level. Out of the 39 stability measures considered, four stability measures (*HMGV*, *RPGV*, *HMRPGV*, *GAI*) are found to be significantly correlated for both datasets. These four measures have an important commonality. They all address both performance and specific types of uncertainty. The first three use BLUP analysis, and *GAI* uses geometric mean. Based on prior studies, *HMGV* is useful when breeders prioritize cultivars that maintain good performance across a range of environmental conditions. *RPGV* focuses on selecting cultivars that are generally superior in performance. *HMRPGV* selects those that perform consistently relative to other cultivars, regardless of the overall environmental yield level. *GAI* selects cultivars that perform consistently across diverse environments by penalizing extreme performance fluctuations and balancing performance in both favorable and unfavorable conditions.

Additional two measures (*Wi_g_*, *Wi_u_*) also have high correlation for only the rapeseed data, and additional five measures have a high correlation for only the sorghum data (*Wi_f_*, *Pi_a_*, *Pi_f_*, *Pi_u_*, *S_6_*). The *Wi* family are based on relative performance of cultivars in different environments, while the *Pi* family measures how close a cultivar’s performance is to the best-performing cultivar across environments. The *S* family on the other hand, executes a rank-based evaluation and identifies those with less variability in ranks across different environments. Thus, 13 out of 39 measures correlate highly with the probabilistic rank for at least one dataset.

**Figure 2:**
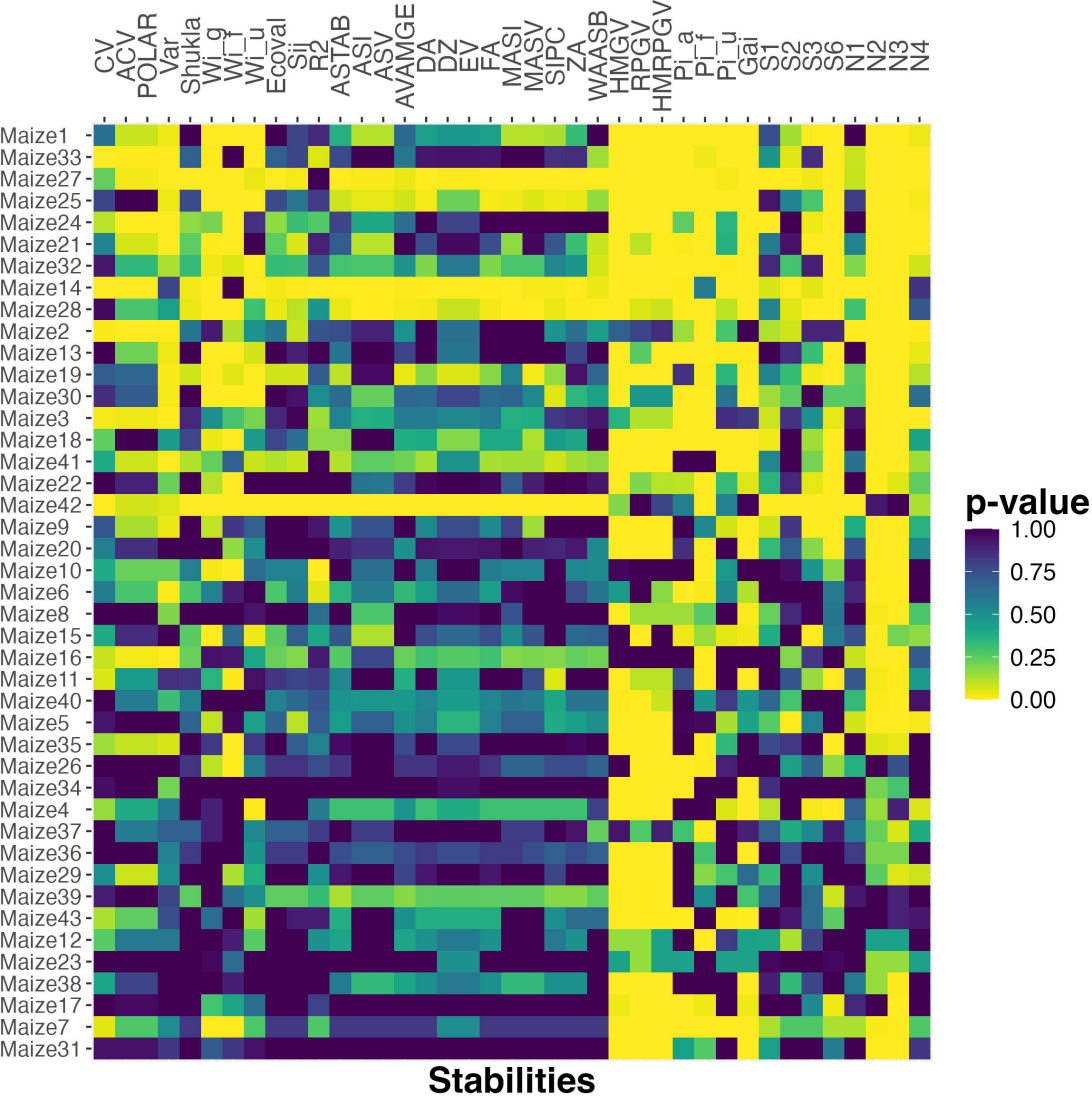
The *p*-value of Spearman’s rank correlation between probabilistic rank and rank according to various stability measures for the maize datasets. The datasets are ordered based on the number of cultivar pairs and the percent of observations that are classified as different.

To see if these observations generalize to a larger testbed, we do the same analysis for all 43 maize datasets constructed based on the G2F data. **Figure 2** plots a heat map of the *p*-values. We observe that the four stability measures that had high correlation for both the smaller datasets (*HMGV*, *RPGV*, *HMRPGV*, *GAI*) also have low *p*-value here for all or almost all of the maize datasets. The *Pi_a_*, *Pi_u_*, *Pi_f_* family of measures also has significantly high correlation with probabilistic ranking here for almost all of the datasets, which is consistent with their high correlation for the sorghum data. Finally, the *Wi_g_*, *Wi_f_*, *Wi_u_* family has high correlation for most of the datasets, similarly supporting their high correlation for the rapeseed data.

The results presented in **Figure 2** reflect the fact that both the probabilistic rank and those from the stability statistics mentioned above, are highly correlated with mean rank. Thus, the high correlation and small *p*-values might simply reflect the dominance of mean in the ranking. In order to address this issue, we next focus on the differences between mean and probabilistic rankings and investigate whether these stability statistics can explain them.

### 3.2 Explaining differences in order

The previous section reports that probabilistic rank has high correlation with both mean rank and the rank according to certain stability measures, especially those that combine mean and stability. In this section we turn to the differences and explore the hypotheses that some stability measures explain the differences between probabilistic and mean order or pairwise comparisons. For the rapeseed and sorghum data there are 2 and 19 pairs of cultivars where the probabilistic order differs from the mean order, respectively.

Recall that the feature selection method that we proposed for this problem is based on the claim that a combination of mean and stability explains when differences occur in mean versus probabilistic order. In particular, for sufficiently large difference in mean all the probabilistic orders become the same as the mean order. The exact cut-off depends on the data. For rapeseed cultivars, the order is the same for all pairs when the absolute value of the mean yield difference is at least 119 Kg/ha. For the sorghum data, the same is true if the mean yield difference is at least 72 kg/ha. Such large yield differences account for 6 out of 15 cultivar pairs for the rapeseed data and 82 out of 153 for the sorghum data. Thus, many pairs are accounted for by large mean differences and the key question is if the stability measures can explain the differences for those pairs where the mean is relatively close. This was the key motivation for our method.

**Figure 3:**
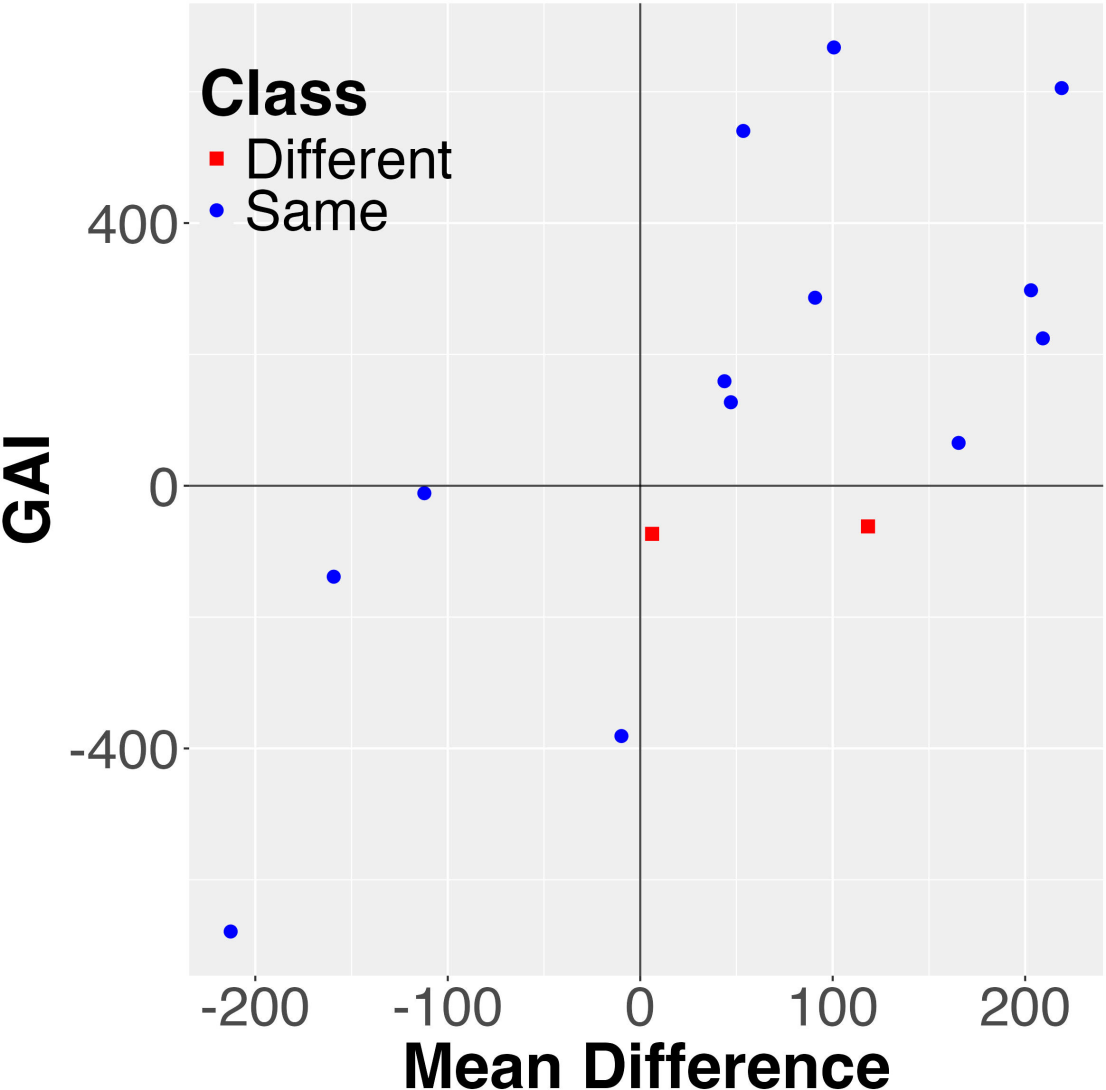
Visualization of the combined mean value and the *GAI* stability measure for explaining differences between probabilistic and mean order for the rapeseed data. Each pair is represented as a data point. Note that all the differences are in one quarter or Region 2, resulting in a *p*-value of zero.

**Figure 4:**
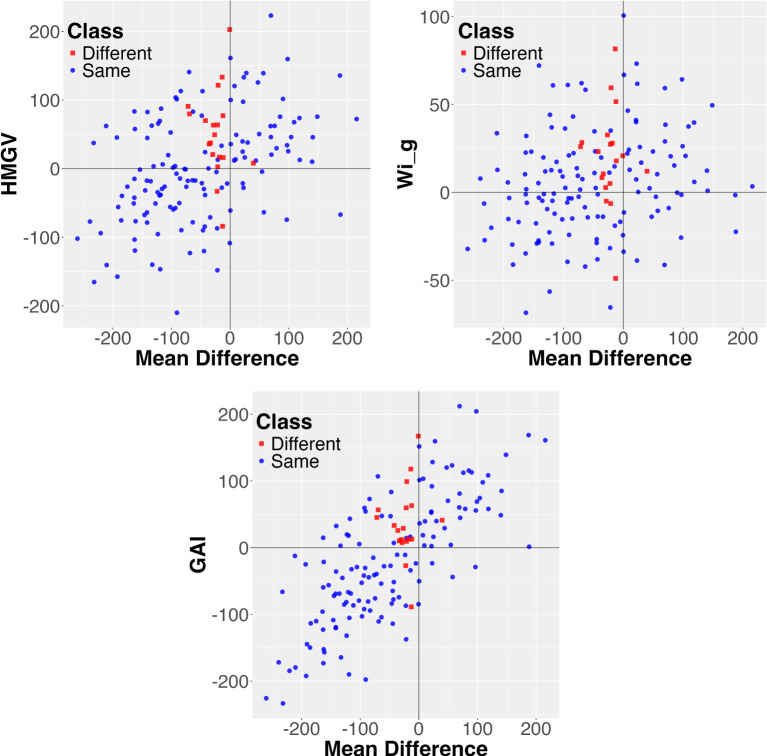
Visualization of the combined mean value and the *HMGV*, *GAI*, and *Wi_g_* stability measures for explaining differences between probabilistic and mean order for the sorghum data. Each pair is represented as a data point. Most of the differences are in Region 2, with only three points in Region 1. Further notice that most differences are close to the *y*-axis, namely the mean difference is small.

For both the rapeseed and sorghum data there are multiple stability metrics that are found significant predictors of the differences. For the rapeseed data it includes *GAI*, *HMRPGV*, *N_2_*, and *RPGV*. And for the sorghum data, there are 25 such stability statistics. Two of the metrics, *GAI* and *HMRPGV*, perfectly explain the differences for the rapeseed data, and this is illustrated in **Figure 3** for the first of those two. We observe that all two of the pairs with different order are in Region 2, while all other pairs are in Region 1, resulting in a perfect classification using just mean and the GAI stability metric (and the corresponding *p*-value is almost zero). For the sorghum dataset no stability measure explains all of the differences, but three explain most of them: *HMGV*, *GAI*, and *Wi_g_*. This is illustrated in **Figure 4**.

**Figure 5:**
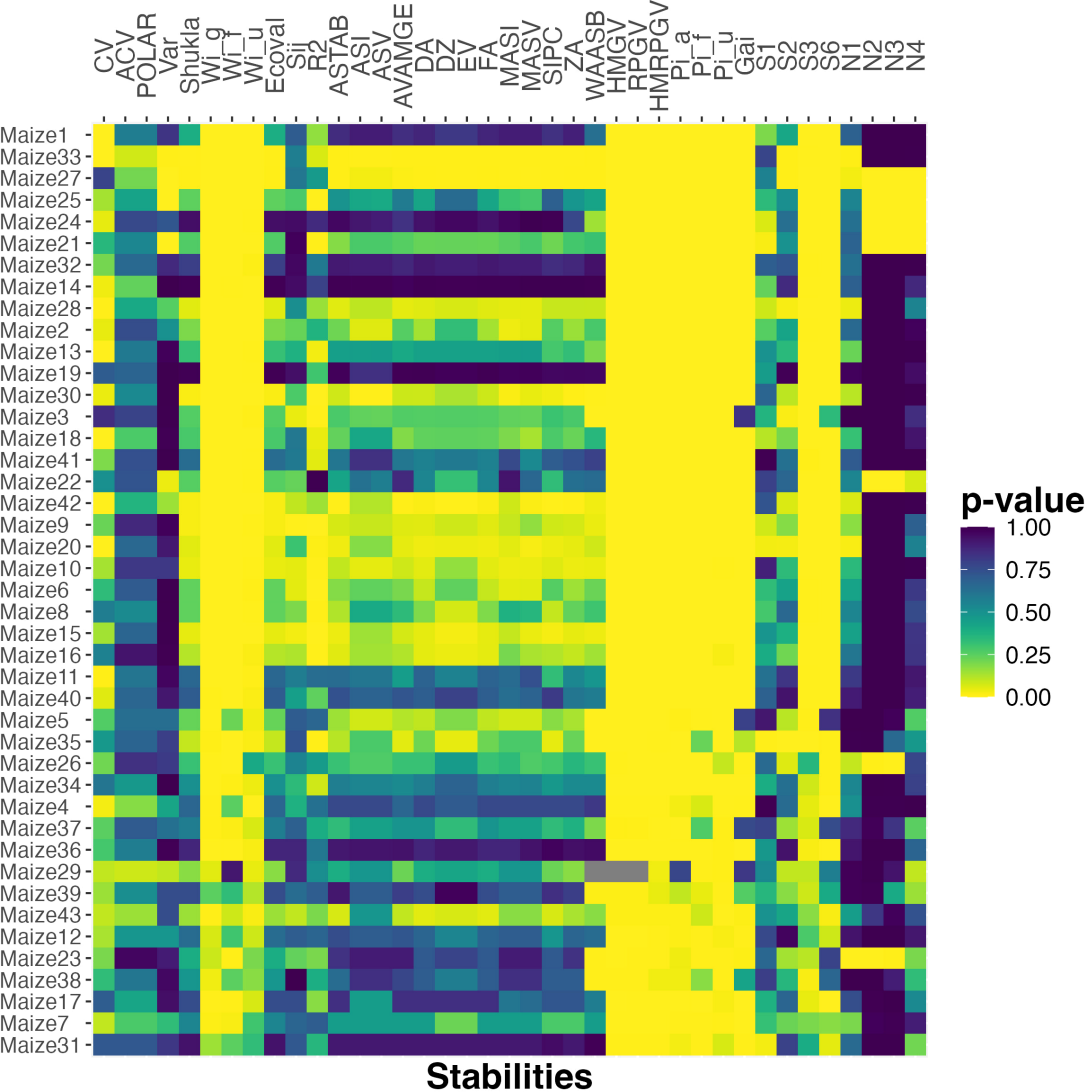
The *p*-value of chi-squared uniformity test between probabilistic rank and rank according to various stability measures for the maize datasets. The datasets are ordered based on the number of cultivar pairs and the percent of observations that are classified as different.

Finally, considering the larger testbed of maize data, **Figure 5** shows a heat map of *p*-values computed by applying this method for all 43 maize datasets using all 39 stability metrics. Relative to the results reported in **Figure 2**, which shows a clear dominance for some stability metrics, **Figure 5** is more noisy since no stability metric captures the differences between mean and probabilistic order well for all 43 datasets. Nonetheless, the same stability metrics perform better in terms of having small *p*-values over a larger number of datasets. In particular, metrics such as *GAI*, *HMGV*, *RPGV*, *HMRPGV*, *N_2_*, that have low *p*-values for the smaller rapeseed and sorghum datasets, also have low *p*-values, and are hence significant predictors, for many or most of the maize datasets.

## 4 Discussion

### 4.1 Key findings

The results show that the mean and probabilistic rank is highly correlated, suggesting that the pairwise orders are usually the same. Some stability measures are also highly correlated with probabilistic rank, and the results show that those tend to be measures that directly combine both mean performance and some type of stability. Specifically, both the harmonic mean and relative performance of genetic value and their combination (*HMGV*, *RPGV*, *HMRPGV*) as well as the geometric adaptability index (*GAI*) are found to have a significantly high correlation for all the data; and two older families of measures: the superiority indices *Pi_a_*, *Pi_u_* and *Pi_f_* of Lin and Binns (1988) on one hand, and the confidence indices *Wi_g_*, *Wi_f_* and *Wi_u_* of Annicchiarico (1997) on the other, have significantly high correlation for some data. This supports the assertion that the probabilistic ranking combines mean performance and stability into a single metric.

When mean and probabilistic order is different, the results show that those differences are often explained by some stability measure. For the simplest data considered (rapeseed), the results show that all of differences could be perfectly explained by a single measure (either *GAI* or *HMRPGV*). This is not the case for the sorghum or maize data, and the measures that best explain the differences varies slightly. For example, *Wi_g_* is one of the three best stability measures to explain differences for the sorghum data even though it did not play a similar role for the rapeseed data. This supports the conclusion that the differences are explained by stability, but depending on the data and possibly other factors, they are best explained by different stability measures, and no measure can simply replace probabilistic comparison and account for all the differences. Also, there does not appear to be a definite pattern in if static or dynamic stability metrics better explains the differences. We speculate that both types of stabilities could be captured depending on the data.

### 4.2 Contributions

This work builds on the work of Bijari (2022) who proposed using bootstrap resampling to estimate the probability that one cultivar performs better than another in a pairwise comparison for random set of target environments. They suggest that this combines mean and stability, which is supported using synthetic data where the magnitude of the G×E effect is controlled. Our main contribution is a systematic evaluation of this claim using real plant breeding data and a comprehensive set of stability measures. Specifically, we propose a classification formulation, and then a feature selection method for this classification problem, that specifically aims to identify which stability measures explain differences between probabilistic and mean order. In addition to these main contributions, we construct new test datasets based on the genome-to-field data that we believe may be useful for testing other new methods in the plant breeding domain.

### 4.3 Limitations

While this work suggest that probabilistic order effectively combines mean and stability based on empirical comparison using select plant breeding data, it is not clear how general this conclusion is in practice. A key limitation is thus that the results reported here do not guarantee that this connection exists for all plant breeding data or establish criteria that plant breeders could use to determine if this connection exist for their trials. Thus, while it suggests that plant breeders may want to consider utilizing probabilistic ranking, it does not theoretically guarantee that the observations made hold for any specific plant breeding trial data and at this point it is thus left to the breeder to determine if this approach works well for their trial data.

Another possible limitation is that the analysis only uses one method for estimating the probability of one cultivar outperforming another, namely an approach based on bootstrap resampling. Other methods could be used, including Bayesian methods, that may provide a more efficient and possibly more precise estimates of the relevant probabilities. Further study is needed to determine the best method(s) for obtaining these probability estimates.

### 4.4 Future research

One direction for further work is motivated by the observation that probabilistic order appears to reflect what might be considered multiple different types of stability (that is, somewhat diverse stability measures have high correlation and explain the differences but none do so for all data). It would be of interest for further investigate how and when different types of phenotype stability is reflected in the probabilistic order. A related future research would be to address the limitations stated above from a plant breeder perspective. Specifically, it would be of significant practical value to identify how characteristics of a plant breeding trial relate to how probabilistic order reflects different stability measures. Finally, this method needs to be tested on many different datasets obtained from a broader variety of crops, with different phenotypes and different growing conditions.

## 5 Conclusion

Probabilistic ranking can be defined as preferring a cultivar that is more likely to perform better in a random set of environments versus the cultivar that performs better on average. Those rankings most often agree, but the claim is that the probabilistic rank combines both mean and stability, so when the mean is sufficiently close the probabilistic rank may prefer the cultivar that is more stable over the one that has better mean. This paper presents a systematic evaluation of this claim using real plant breeding data and a comprehensive set of stability measures. The conclusion of the analysis is that based on the results presented here, differences in mean and probabilistic ranking are in fact explained by differences in stability, supporting the claim that probabilistic ranking effectively combines mean and stability into a single measure. Based on this, plant breeders may consider probabilistic ranking as an alternative to mean-based ranking supplemented by the use of stability measures.

## Data Availability

The original contributions presented in the study are included in the article/supplementary material. Further inquiries can be directed to the corresponding author.
